# Supplementary material for: Road to Metastasis: The TWEAK Pathway as a Discriminant between Metastasizing and Non-Metastasizing Thick Melanomas
Source: Int J Mol Sci. 2021 Sep 29;22(19):10568. doi: 10.3390/ijms221910568 (PMC8508767; doi:10.3390/ijms221910568)
Supplement: Supplementary file 1 [file ijms-22-10568-s001.zip › supplementary figure S1- Volcano plot Leedsdatabase.pdf]

Supplementary Figure S1: Volcano plot Leedsdatabase

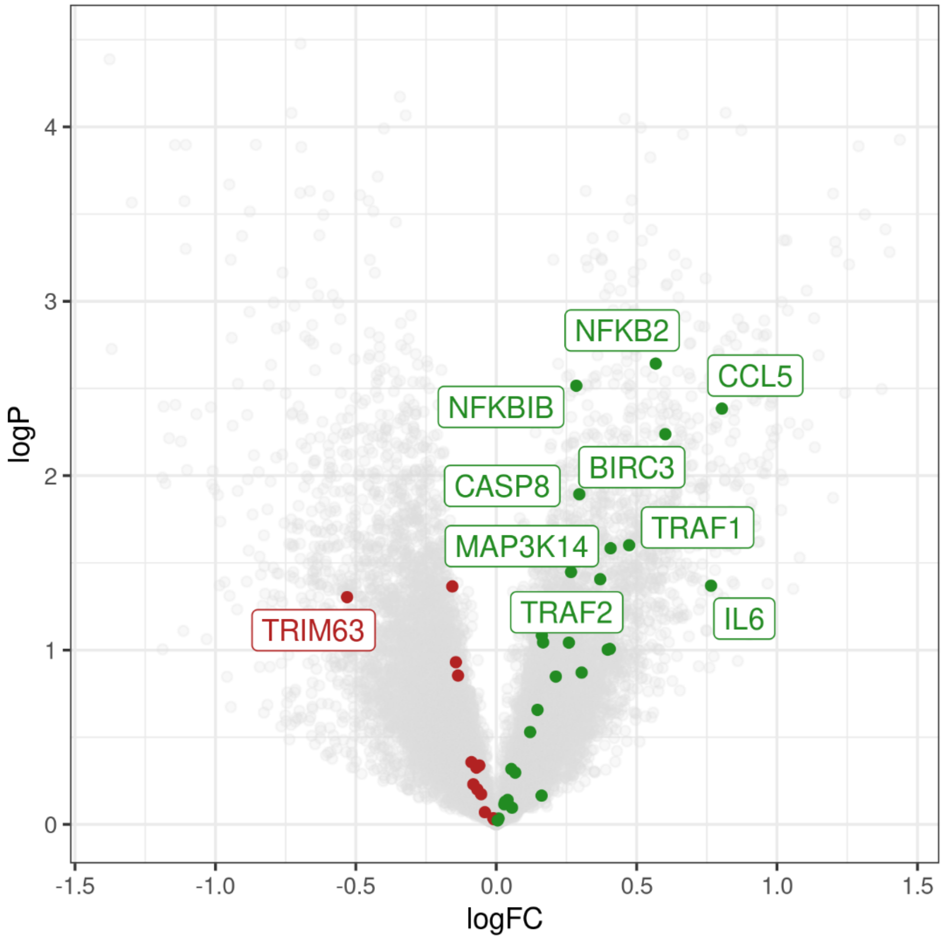

Volcano Plot for differentially gene expression in the Leeds database : red boxes are genes with higher expression in thick M+ (left) , and green boxes are genes with higher expression in thick M- (right) B
