# Supplementary material for: Road to Metastasis: The TWEAK Pathway as a Discriminant between Metastasizing and Non-Metastasizing Thick Melanomas
Source: Int J Mol Sci. 2021 Sep 29;22(19):10568. doi: 10.3390/ijms221910568 (PMC8508767; doi:10.3390/ijms221910568)
Supplement: Supplementary file 1 [file ijms-22-10568-s001.zip › Supplementary table S1a and S1b The multivariate logistic regression analysis in thick CM-Methods.pdf]

Supplementary Table S1a: The multivariate logistic regression analysis for the association between development of metastasis within five years and clinico-pathological parameters (UZ database)

Prognostic variables										
			Univariate analysis				Mutlivariate analysis			
				95% C.I.for OR				95% C.I.for OR		
**Variable**	**Contrast**	**N** *(total)*	**OR**	**Lower**	**Upper**	***P*-value**	**OR**	**Lower**	**Upper**	***P*-value**
		140								
**Gender**	*Male vs Female*		1,37	0,70	2,68	0,36	1,30	0,57	2,96	0,53
**Age**	*≤ 45 yrs vs > 45 yrs*		1,14	0,52	2,49	0,75	1,42	0,58	3,47	0,45
**Breslow thickness**	*2 - 4 mm vs ≥ 4 mm*		0,84	0,40	1,74	0,63	1,00	0,31	2,00	0,54
**Ulceration**	*Yes vs No*		0,63	0,31	1,30	0,21	1,00	0,32	2,00	0,46
**Mitosis**						0,97				1,00
	0		0,00	0,00	.	1,00	0,00	0,00	.	1,00
	1-6		0,86	0,12	6,43	0,89	0,78	0,09	6,94	0,83
	7-10		0,65	0,08	5,21	0,69	0,78	0,08	7,60	0,83
	11-20		0,73	0,08	6,31	0,77	0,86	0,08	8,90	0,90
	>20 (ref)									
**Localisation**						0,38				0,65
	Trunk		1,26	0,57	2,77	0,57	1,00	0,58	4,00	0,44
	Head & neck		0,59	0,23	1,53	0,28	1,65	0,36	7,00	0,52
	Extremities (ref)									
**Histopathological prognostic variables**										
**Subtype**						0,58				0,83
	NM		0,77	0,38	1,56	0,47	1,00	0,50	3,00	0,72
	AM		1,43	0,40	5,15	0,58	2,00	0,35	7,00	0,56
	SSM (ref)									
**TIL**						0,47				0,55
	Brisk		0,44	0,08	2,25	0,32	0,00	0,06	2,00	0,30
	No TILs		1,30	0,55	3,08	0,55	1,00	0,43	3,00	0,78
	Non-brisk(ref)									
**Vascular invasion**	*Yes vs No*		0,00	0,00	.	1,00	0,00	0,00	.	1,00
**Solar damage**						**0,05**				0,07
	1		11,60	1,44	93,56	**0,02**	1,34	0,57	3,19	0,50
	2		17,33	2,10	142,78	**0,01**	0,58	0,14	2,43	0,46
	3		7,78	0,78	77,93	**0,08**	0,05	0,00	0,64	0,02
	Solar damage (ref)									
**Nevus**	*Yes vs No*		2127041876,42	0,00	.	1,00	960414224,80	0,00	.	1,00

Abbreviations Supplementary Table S1a: yrs: years; mm: millimetres; ref: reference; NM: nodular melanoma; AM: acral melanoma; SSM: superficial spreading melanoma; TIL: tumour infiltrating lymphocyte; C.I.: confidence interval; OR: Odds ratio. The multivariable analyses consisted of two steps. In a first step, results were reported from a multivariable model, with age, gender, stage, localization and subtype as predictors. In a second step, this model is extended with each of the variables.

Supplementary Table S1b: The multivariate logistic regression analysis for the association between development metastasis within five years and clinico-pathological parameters (Leeds database)

**Prognostic variables**										
		**Univariate analysis**					**Mutlivariate analysis**			
			**95% C.I.for OR**					**95% C.I.for OR**		
**Variable**	**Contrast**	**OR**	**Lower**	**Upper**	***P*-value**	**N** *(total)*	**OR**	**Lower**	**Upper**	***P*-value**
						141,00				
**Gender**	*Male vs Female*	1,02	0,29	3,55	0,98		1,61	0,64	4,06	0,31
**Age**	*≤ 45 yrs vs > 45 yrs*	2,19	0,61	7,85	0,23		1,49	0,51	4,34	0,47
**Breslow thickness**	*2 - 4 mm vs ≥ 4 mm*	1,30	0,38	4,52	0,68		1,61	0,64	4,06	0,31
**Ulceration**	*Yes vs No*	1,09	0,33	3,63	0,89		1,16	0,47	2,86	0,75
**Mitosis**					0,67					0,42
	0	0,08	0,00	3,12	0,18		0,06	0,00	1,71	0,10
	1-6	0,71	0,07	7,10	0,77		0,51	0,06	4,20	0,53
	7-10	0,48	0,04	5,94	0,56		0,35	0,03	3,70	0,38
	11-20	0,70	0,05	9,49	0,79		0,92	0,08	10,82	0,95
	>20 (ref)									
**Localisation**					0,54					0,77
	Trunk	0,49	0,14	1,71	0,26		0,71	0,28	1,78	0,47
	Head & neck	139147603,40	0,00	.	1,00		177850441,42	0,00	.	1,00
	Extremities (ref)									
**Histopathological prognostic variables**										
**TIL**					0,41					0,29
	Brisk	159575793,84	0,00	.	1,00		266470077,17	0,00	.	1,00
	No TILs	4,38	0,51	37,68	0,18		5,28	0,67	41,39	0,11
	Non-brisk(ref)									
**Vascular invasion**	*Yes vs No*	1,86	0,36	9,57	0,46		1,71	0,48	6,12	0,41

Abbreviations **Supplementary Table S1b**: yrs: years; mm: millimetres; ref: reference; NM: nodular melanoma; AM: acral melanoma; SSM: superficial spreading melanoma; TIL: tumour infiltrating lymphocyte; C.I.: confidence interval; OR: Odds ratio. The multivariable analyses consisted of two steps. In a first step, results were reported from a multivariable model, with age, gender, stage, localization and subtype as predictors. In a second step, this model is extended with each of the variables.

Materials and methods

We retrospectively selected 140 patients from UZleuven database who met the following inclusion criteria: (1) >18 years, (2) CM with Breslow ≥ 2mm, (3) 5 year follow up; we excluded desmoplastic, nevoid and Spitzoid melanomas. Clinical and pathological characteristics were analyzed against a defined outcome (thick metastasizing and thick non-metastasizing CM). Each melanoma sample of our UZLeuven cohort was reviewed by two experienced pathologists (J. J. van den Oord and Francesca Bosisio). Available clinical data included age (<40, 40-59 years, >=60 years), sex, site (extremity, head and neck, trunk), subtype, Breslow thickness, ulceration, mitotic rate and AJCC stage at diagnosis, tumor infiltrating lymphocytes (TIL), Vascular invasion, solar damage and adjoining nevus (1-2)). Univariate analysis was employed to examine the individual effects of each clinical and pathological parameter on outcome (thick metastasizing CM and thick non-metastasizing CM). Multivariate logistic regression was performed to identify clinical and pathological predictors of outcome in CM, which had been adjusted by the known prognostic variables (sex, age, Breslow thickness, ulceration, mitotic rate and localization). Correction for multiple testing was conducted by implementing the Bonferroni method. For the validation part we extracted data of melanoma patients from the Leeds Melanoma Cohort (3) in accordance with the inclusion and exclusion criteria of the UZleuven database.

Some of the parameters included in the analysis showed extreme odds ratio (OR) and confidence interval (CI), probably due to absence of events in certain categories or due to small sample size of the parameter in a specific category eg vascular invasion (only one sample with vascular invasion), mitosis, nevus, site, TIL. Since these parameters are of interest to our study, we have included these parameters in the analysis.

1. Balch CM, Gershenwald JE, Soong SJ, Thompson JF, Atkins MB, Byrd DR, et al. Final version of 2009 AJCC melanoma staging and classification. J Clin Oncol. 2009;27(36):6199-206.

2. Gershenwald JE, Scolyer RA, Hess KR, Sondak VK, Long GV, Ross MI, et al. Melanoma staging: Evidence-based changes in the American Joint Committee on Cancer eighth edition cancer staging manual. CA Cancer J Clin. 2017;67(6):472-92.

3. Thakur R, Laye JP, Lauss M, Diaz JMS, O'Shea SJ, Pozniak J, et al. Transcriptomic Analysis Reveals Prognostic Molecular Signatures of Stage I Melanoma. Clinical Cancer Research. 2019;25(24):7424-35.
